# Effects of nutritional cues on the duration of the winter anovulatory phase and on associated hormone levels in adult female Welsh pony horses (Equus caballus)

**DOI:** 10.1186/1477-7827-9-130

**Published:** 2011-09-29

**Authors:** Juan Salazar-Ortiz, Sylvaine Camous, Christine Briant, Lionel Lardic, Didier Chesneau, Daniel Guillaume

**Affiliations:** 1 INRA, UMR85 Physiologie de la Reproduction et des Comportements, F-37380 Nouzilly, France; 2 CNRS, UMR6175 Physiologie de la Reproduction et des Comportements, F-37380 Nouzilly, France; 3 Université François Rabelais de Tours, F-37041 Tours, France; 4 IFCE, F-37380 Nouzilly, France; 5 INRA, UMR1198 Biologie du Développement et Reproduction, F-78352 Jouy-en-Josas, France; 6 ENVA, F-94704 Maisons Alfort, France

## Abstract

**Background:**

Mares have an annual reproductive rhythm, with a phase of inactivity in midwinter. The aim of this study was to determine the impact of food restriction on physiological and metabolic hallmarks of this rhythm.

**Methods:**

Over three successive years, 3 groups of 10 mares were kept under natural photoperiod. A 'well-fed' group was fed to maintain the mares in good body condition; a 'restricted' group received a diet calculated to keep the mares thin and a 'variable' group was fed during some periods like the 'restricted' group and during some other periods like the 'well-fed' group, with the aim of mimicking the natural seasonal variation of pasture availability, but a few months in advance of this natural rhythm.

**Results:**

Winter ovarian inactivity always occurred and was long in the restricted group. In contrast, in the 'well-fed' group, 40% of mares showed this inactivity, which was shorter than in the other groups. Re-feeding the 'variable' group in autumn and winter did not advance the first ovulation in spring, compared with the 'restricted' group. Measurements of glucose and insulin concentrations in mares from the 'restricted' group during two 24 h periods of blood sampling, revealed no post-prandial peaks. For GH (Growth hormone), IGF-1 and leptin levels, large differences were found between the 'well-fed' group and the other groups. The glucose, insulin, GH and leptin levels but not melatonin level are highly correlated with the duration of ovulatory activity.

**Conclusions:**

The annual rhythm driven by melatonin secretion is only responsible for the timing of the breeding season. The occurrence and length of winter ovarian inactivity is defined by metabolic hormones.

## Background

In horses, the natural breeding season (without light treatment) is centred on the longest day of the year (20^th ^June in the northern hemisphere). The breeding season begins in spring when duration of daylight, ambient temperature and food availability increase. Consequently, a peak of births occurs 11 months later, at the end of May. For wild mares, winter ovarian inactivity is an important adaptation to the environment. Due to regulations of horses' races and competitions, births early in the year gave benefits for races or sports [1,2]. Furthermore, if mares can be mated early in the year, the number of cycles, and then the success of pregnancy at the end of the year, increases [3].

Several authors have suggested that nutrition, and particularly energy intake, has an effect on the onset of ovulatory activity [4-8]. A proportion of adult mares (> 5 years old) ovulated continuously throughout the year [9,10]. However, mares that had nursed a foal during the previous summer, young mares (< 4 years old) and lean mares all showed winter ovulatory inactivity [11-13]. Conversely, mares that did not show winter ovulatory inactivity had a higher percentage of body fat than mares with inactivity [14]. These data indicate that body weight, fat reserves and the availability of food are important factors affecting the pattern of seasonal ovulatory inactivity and suggest that nutrition may be one factor that interacts with photoperiod to determine the precise onset and duration of ovarian inactivity in the mare.

The main site of action of nutrition on the ovarian cycle in cows [15,16], gilts [17] and mares [10,18] appears to be the hypothalamus, and the mechanism probably has multiple components including insulin, leptin, growth hormone (GH) and insulin-like growth factors (IGFs). It has been shown that induced hypoglycaemia decreases secretion of gonadotrophins and results in anovulation in different species including mares [14,19-22].

Leptin, an adipocytokine, regulator of food intake and satiety [23], informs the brain on body condition and adiposity, and probably interacts with photoperiod to impacts reproduction. In horses, the plasma concentration of leptin is strongly correlated with body condition score [24]. In human blood, this protein binds to five 'serum leptin-interacting proteins' [25]. In adult castrated rams, a specific receptor present at the blood brain barrier seasonally modulates the active transport of leptin from the blood to the brain [26]. Injection into the ovine cerebrospinal fluid strongly affects GnRH, LH and GH secretion [27,28].

The GH and IGF systems influence reproduction [29]. GH affects the ovary by enhancing the gonadotrophin responsiveness of this organ [30]. IGF-I is the key mediator of most of the actions of GH and exerts negative feedback on secretion [31]. In horses, plasma IGF-I concentrations are not influenced by time of day or by exercise, but are dramatically increased by exogenous GH [32].

In sheep [33], goats [34], deer [35] and horses [36-38], the annual reproductive season is synchronized by photoperiod through melatonin secretion. In mares, the date of first ovulation can be advanced by about two months by exposure to an artificial photoperiod in winter [36,37,39]. In anovulatory mares during winter, treatment with exogenous melatonin suppresses the stimulatory effect of artificial long photoperiods [40,41]. Conversely, the effect of photoperiod or exogenous melatonin on the time of the last ovulation in autumn is controversial. In adult mares, melatonin treatment fails to change the date of the last ovulation [41,42]. In horses [12,14,39,41,43-48], as in ewes [49], different experimental approaches indicate that the annual reproductive rhythm has a strong endogenous component. The phase of this annual endogenous rhythm is regulated by photoperiod through mediator melatonin.

The hypotheses, tested in this experiment study are 1) body condition induced by feed intakes affects the occurrence and duration of seasonal anoestrus in mare, 2) a change in feed intake can modify the date of the first ovulation of the year, 3) this change affects daily patterns of plasma glucose, insulin, melatonin, GH, IGF-1 and leptin.

## Methods

### Animals and experimental conditions

The experiment was conducted in the course of 3 years, from August to August, on Welsh pony mares (*Equus caballus*) of the experimental herd from the National Institute of Agronomic Research (INRA, latitude 48°N) in accordance with national animal ethics requirements (French Ministry of Agriculture, Fishing and the Countryside [A37801] and animal experimentation permit 3706). The same 30 adult mares were used all along the experiment. They had not nursed a foal the previous year. At the beginning of the experiment, they were 7.2 ± 0.5 years old (Mean ± SEM), weighed 301 ± 8 kg and had a body condition score of 3.5 ± 0.2 with the French scoring system [50] which combine visual examination and body palpation (0 emaciated and 5 obese) and which approximately corresponded to the scoring system previously developed [51]. They were assigned, for all the experiment, to 3 groups using a stratified randomization: well fed (WF), restricted (R) and variable (V). The groups were stratified by age, body weight and paternal origin. Ponies were kept in groups of 5 in 25 m^2 ^boxes. They had straw *ad libitum *for litter and forage. They had free access to water and to a salt mineral lick; during the day they also had access to a 70 m^2 ^paddock with sand. Normal husbandry procedures, worming and vaccine treatments were followed. All mares were kept under natural photoperiod, which in this latitude ranges from 8 hours of light at the winter solstice to 16 hours of light at the summer solstice. One mare from the WF group was excluded from the experiment due to an accidental fracture.

### Experimental design

**Figure 1:**
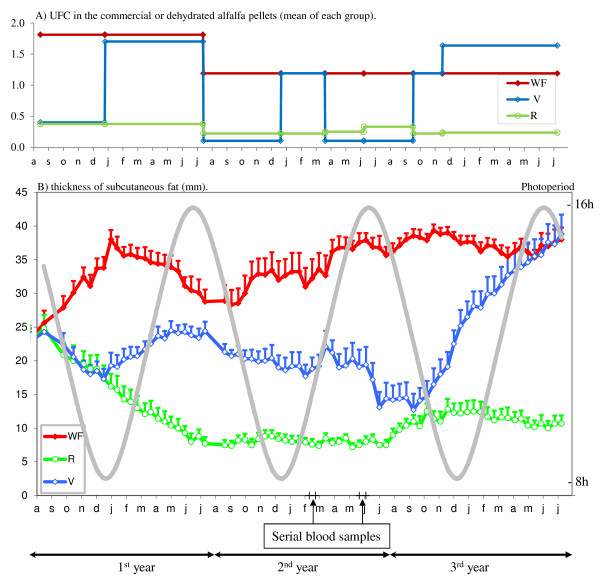
**Fat thickness fluctuations during the three-year study**. Part A: Experimental variation of average of feed intake in WF, R and V groups expressed in Horse Feed Unit (UFC) contained in the commercial or dehydrated alfalfa pellets. Part B: Fat thickness fluctuations (means ± S.E.M), in WF, R and V groups during the three-year study under natural photoperiod conditions. The WF and R groups received a diet supplying approximately 100% and 50% of energy requirements for maintenance, respectively. The V group was fed in order to mimic variations of energy availability in natural grasslands, but about 4-5 months in advance of phase relative to the beginning of natural availability of grass in pastures. The arrows indicate the date when the blood samples were collected each hour during two 24 h periods.

Quantities of pellets (composition in Table 1) estimated for each group were given in the morning at around 07:30 h and in the evening at around 16:00 h. During the first year, the diet was extrapolated from values obtained for saddle horses [52,53]. In the second year, the diets were corrected for the energy requirements of ponies [54]. The variation of the mean quantity of energy expressed in Horse Feed Unit (UFC) given by these pellets for each group are presented on Figure 1.

**Table 1:** Composition of feeds used for experimental mares.

	Wheat straw	Commercial Pellets	Dehydrated alfalfa pellets
UFC	0.26/kg	0.71/kg	0.51/kg
MADC	0 g/kg	83.8 g/kg	90 g/kg
Ca	4.4 g/kg	10 g/kg	22 g/kg
P	0.7 g/kg	4 g/kg	2.2 g/kg
Mg	0.6 mg/kg	30.5 mg/kg	2.0 mg/kg

UFC = Horse Feed Unit [50]

MADC = Horse Digestible Crude Protein [50]

*(a) The WF group: *In year 1, the mares in the WF group received 2.5 kg/day of commercial pellets (Table 1). At the end of the year, the body weight of the pony mares of the WF group kept on increasing so that the quantity of pellets was reduced to 1.6 kg/day during the 2^nd ^and 3^rd ^years.

*(b) The R group: *The mares received a diet calculated to reduce their weight. The diet was then adjusted individually to keep the mares lean. The mares in this group received on average 0.74 kg/day of dehydrated alfalfa pellets which were chosen to provide a satisfactory protein/energy ratio. This diet provided approximately 50% of energy requirements calculated on the mares' body weights measured at the beginning of the experiment.

*(c) The V group: *The mares in the V group were fed in a manner that mimicked the normal seasonal variations of available energy in a natural pasture under temperate latitudes but with a phase advance of 4-5 months, with the aim to advance the start of ovulatory activity. These mares were fed either as for the WF group or the R group (Table 2 and Figure 1). The last year of the experiment the feed intake was intensified with the aim to reinforce the effect on the first ovulation.

**Table 2:** Calendar of mares' feeding and quantity of commercial or alfalfa pellets in group V.

	Restricted period		Well-fed period	
	**Beginning** **of the period**	**Alfalfa pellets** **(kg/day)**	**Beginning** **of the period**	**Commercial pellets** **(kg/day)**
Year 1	13 August	0.74	28 December	2.4
Year 2	21 July	0.23	30 December	1.7
Year 3	1 April	0.23	2 Oct. and 24 Dec.	1.7 and 2.4

### Body weight, fat thickness and body condition score

Body weight (BW) and thickness of subcutaneous fat were measured every 15 days in the early afternoon. Thickness of subcutaneous fat was measured with an Akola SS-210 DX ultrasound equipped with a 5-megahertz linear-array transducer. Measurements were taken on the middle of the croup, around 5 cm lateral to the midline. Body condition score (BCS) was assessed during the years 2 and 3, every 15 days, using the French scale [50].

### Ovulatory activity

From the beginning of the experiment, blood samples were collected by venepuncture of the left jugular vein twice a week into heparinized tubes (Vacutainer^® ^Vecton-Dickinson). The plasma was recovered by centrifugation and stored at -20°C. Plasma progesterone was assayed with a previously validated radio-immuno-assay (RIA) [55]. Mares were considered cyclic if plasma progesterone concentration was greater than 1 ng/ml in at least two successive samples. They were classified as being in anoestrus if their progesterone levels were below 1 ng/ml for more than 4 consecutive weeks. The last date when the progesterone concentration was above 1 ng/ml in the last ovulatory cycle of the breeding season was deemed as the start of anovulatory period, and the first date when progesterone was greater than 1 ng/ml in the first ovulatory cycle of the new breeding season was deemed as the end of anovulatory period

### Determination of plasma concentrations of glucose, insulin, melatonin, GH, IGF-1 and leptin

In year 2 of the experiment, when the body weights of the R and WF groups were stabilised and 1 month before and 2 months after the change of feed intake of the V group, blood samples were collected and processed as above, every hour during two 24 h sampling windows, one in winter (5 mares in each groups on 19^th ^February and the other mares on 3^rd ^March) and one in spring (with the same mares repartition: 3^rd ^and 17^th ^June). During darkness, the mares were tethered and samples were collected using a red light of low intensity (< 1 lux at 20 cm). The different hormones, for all these samples, are quantified in a single assay.

#### Melatonin

Melatonin levels were determined by RIA using a specific anti-melatonin antiserum obtained in rabbit [56], adapted for mare plasma [40]. The minimum level of detection was 5 pg/ml. The intra-assay coefficient of variation on samples containing 35 pg/ml of melatonin was 19%.

#### Glucose

Glucose concentrations were measured by the glucose oxidase method [57] in a Coulter glucose analyzer (Beckman ^®^, Palo Alto, CA, USA). In this method, 10 μl of sample were added to glucose oxidase in a well containing an oxygen-sensitive electrode. The rate of oxygen consumption is directly proportional to the glucose concentration. For a standard of 450 mg/dl, the stated error is ± 3%.

#### Insulin

An insulin RIA was developed using pig insulin for standards and iodination and a guinea pig antibody to porcine insulin (P-Insulin I5523, anti-pig insulin I-8510, SIGMA Chemical, St Louis MO, USA). Pig and equine insulin differ only by one amino acid in the A chain. The antibody was used at a final dilution of 1/80,000 with approximately 20,000 CPM of pig ^125^I insulin. To separate bound and free, a 2^nd ^antibody against guinea pig gamma globulins raised in the horse was used. To make standards, P-insulin, ranging from 0.025 to 800 ng/ml was dissolved in a mare's plasma. This mare was previously kept without food during 1 day and her plasma treated with charcoal. A 5-parameter logistic curve was used to calculate the sample values. The minimum level of detection was 1.25 pg/ml and the intra-assay coefficients of variation for plasma samples containing 1.4 and 2.7 ng/ml of equine insulin were 7.2% and 5.8% respectively.

#### Growth hormone

A homologous RIA assay developed in our laboratory was similar to a method previously described for ePRL [58].

The first antibody was obtained in rabbit against equine recombinant GH (erGH) (EquiGen-5^® ^BresaGen Limited, 38 Winwood Street, The Barton, SA 5031, Australia). A highly purified eGH (AFP71128 National Hormone Peptide Program, Harbor-UCLA Medical Center, Torrance, CA, USA) was used for radioiodination and standards. Eleven standards of eGH dissolved in hypophysectomized mare plasma ranging from 0.125 to 1000 ng/ml were used with a 5-parameter logistic curve to calculate the sample value. The percentage cross-reaction was estimated as the ratio of the abscissa of the inflection point of the 5-parameter logistic function calculated between different standards. Using the curve obtained with erGH as reference, the percentage cross-reactions with eGH and ePRL were 60% and 8% respectively. No other cross-reaction was found with other equine hormones. The dilution tests give a determination coefficient of the regression of 99.67%. The minimum level of detection was 1 ng/ml. All samples were measured in the same assay, in duplicate. Old mare plasma and triplicate blood samples were routinely used every 100 samples, to calculate the intra-assay coefficients of variation. The eGH level in the old mare plasma was never detectable. The coefficients of variation for plasma samples containing 85, 55 and 30 ng/ml of eGH were 5.2, 9.6 and 7.4%, respectively.

#### IGF-1

A commercial kit validated for human IGF-1 (CISbio international BP 32 F91192 Gif sur Yvette Cedex France) was used. To summarize: this kit is a "sandwich" immunoradiometric assay. Two monoclonal antibodies were prepared against two different antigenic sites of IGF-1. The first was coated into the assay tube and the second was radiolabeled with iodine 125. Before the assay, the IGF-1 of sample was separated from IGFBPs by dissolution in an acid solution and saturation with a recombinant human IGF-2. To validate the kit for eIGF-1, dilution tests of blood sample from a fat mare in buffer or in plasma from a thin mare were done. The determination coefficients of the regression are 99 and 92% respectively. The intra-assay coefficients of variation, estimated with 2 plasma samples assigned each 100 samples and containing 33 and 378 ng/ml of hIGF-1 were 4.3 and 8.4%, respectively.

#### Leptin

Plasma samples were analyzed with a homologous double-antibody RIA developed in our laboratory, similar to the one previously described for ovine leptin [59]. This assay used a primary antiserum obtained from goats against recombinant equine leptin (a gift from A. Gertler, the Hebrew University, Rehovot, Israel). Standard (0.75 to 20 ng recombinant equine leptin/ml) or unknown samples diluted in PABET buffer [24] were incubated for 24 h at room temperature with equine leptin antiserum (1:2,000 initial dilution). The next day, equine ^125^I-leptin was added to each tube and the incubation continued for 24 h. A rabbit antiserum against sheep gamma globulins was used to precipitate the complex made with 1^st ^antibody and leptin. The intra- and inter-assay coefficients of variation were about 10% and 13%, respectively. No cross-reaction of the equine leptin antiserum was observed with recombinant rabbit, ovine and human leptins. Serial dilutions of equine pooled plasma gave a linear displacement curve after log logit transformation (slope: -1.47, r^2 ^= 0.96) that was parallel to those of the standard preparation (slope: -1.56, r^2 ^= 0.95). The limit of detection was 1 ng/ml.

### Statistical analysis

To compare the number of mares with ovulatory inactivity between the 3 groups, a non-parametric Khi2 Pearson exact test [60] was used; when the global comparison was significant, the comparisons of the groups 2 × 2 were done with unilateral exact Fisher test [61]. To compare the number of luteal phases during each year in each group, a non-parametric ANOVA with general score test was used; when the global comparison was significant the comparisons of the groups 2 × 2 were done with a bilateral exact permutation test [62]. For the 2 × 2 comparisons the Bonferroni correction was used. These non-parametric tests were done with StaXact^® ^Cytel (Software Corporation, 236 Cambridge, MA 02139, USA).

All the other parametric statistical tests were carried out using SAS software (SAS Institute Inc., Cary, NC 27513, USA). Body weight, fat thickness and BCS were compared by analysis of variance (ANOVA) using a repeated measures under the general linear model (GLM) procedure [63]. For the analysis, each season (season, defined with solstice and equinox) was treated as a separate period.

To compare the dates of the start, the end and the middle of anoestrus between groups and between years, these dates were expressed as the number of days from the summer or the winter solstice and log-transformed. These data and the duration of anoestrus (in days), were examined by ANOVA using a repeated measures model under GLM procedure.

The daily melatonin, glucose, insulin, GH, IGF-1 and leptin patterns (after log transformation except for IGF-1) were examined by ANOVA using the GLM procedure [63]. The analysis tested 3 factors: treatment (WF, R and V), season (winter and summer) and mares nested under treatment. For melatonin and leptin, the analysis tested the effect of the light-dark cycle using 4 levels (light phase in winter or summer, dark phase in winter or summer). For glucose, insulin and leptin, a final analysis tested the effect of the postprandial peak, using 8 levels (for each season, the light phase was divided into 3 phases of 4 hours, the first phase starting after the first food distribution and the night phase of 12 h).

The number of mares with winter ovarian inactivity was unbalanced between treatment groups. Therefore, to correlate these data with body weight, fat thickness, BCS, hormone and glucose levels, we compared the first phase without progesterone (included the follicular phase for mares whose cycles did not stop in winter and the anovulatory winter phase). Correlation coefficients were estimated using Pearson's correlation method.

The results are systematically presented as arithmetic mean ± SEM.

## Results and discussion

### Body weight, body condition score and fat thickness

The mean weights did not differ between the groups at the start of the experiment (301 ± 8 kg). In the WF group, the mares gained 22.7 ± 8.2 kg in the 1^st ^year and were stabilized for the remaining 2 years. Mares in the R group lost 66.2 ± 6.1 kg during the 1^st ^year and then were stabilized at around 228.5 ± 1.5 kg. They were significantly lighter than the mares in the WF group. The V group mares fluctuated between the two other groups (p < 0.0001). The lowest correlation, observed between fat thickness and body weight, was, during the 1^st ^autumn, 0.53 (p < 0.01) and at the same season, between fat thickness and BCS was 0.90 (p < 0.0001). The two by two correlations between, body weight, body condition score and fat thickness were all highly significant, so we selected only one, fat thickness, for the analysis (Figure 1).

### Anovulatory period

**Figure 2:**
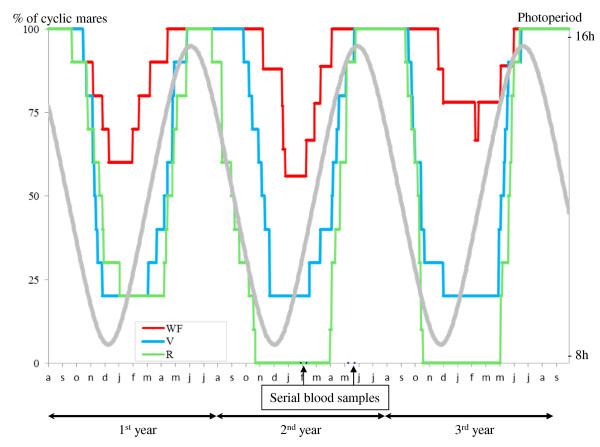
**Percentage of cyclic mares**. The 3 groups were maintained under natural photoperiodic conditions and fed with 3 different diets. The WF and R groups received a diet supplying approximately 100% and 50% of energy requirements for maintenance, respectively. The V group was fed in order to mimic variations of energy availability in natural grasslands, but about 4-5 months in advance of phase relative to the beginning of natural availability of grass in pastures.

The percentages of cyclic mares are shown in Figure 2. The main parameters of ovulatory inactivity are summarized in Table 3. During the breeding season, all the mares had at least one ovulatory cycle. The number of mares with winter ovulatory inactivity differed significantly between the 3 groups in each winter (p < 0.001). Only, 44% of the WF mares had a period of seasonal anovulation while for the R group the figure was 80% on the 1^st ^winter and 100% on 2^nd ^and 3^rd ^winters. In the WF group the same 4 mares showed an anoestrus period in each winter. In the V group, 20% of the mares never showed winter ovulatory inactivity, while the other 80% had a period of anovulation on each winter.

**Table 3:** Mean dates ± S.E.M. (in days) of beginning, end and durations of ovulatory inactivity in mares kept under the 3 experimental diets and under natural photoperiod.

		Ovarian inactivity 1^st ^winter	Ovarian inactivity 2^nd ^winter	Ovarian inactivity 3^rd ^winter	Mean duration (days) (2) (3)	
WF	Beginning	21 Nov ± 12	31 Dec ± 10	14 Jan ± 25		
	End	22 March ± 17	28 March ± 12	26 April ± 23		
	Duration (1)	119 ± 37 (4/10)	87 ± 17 (4/9)*	103 ± 13 (3/9)	103.0	40.4
R	Beginning	21 Nov ± 11	22 Sep ± 10	27 Oct ± 4		
	End	21 May ± 7	15 May ± 6	21 May ± 4		
	Duration (1)	180 ± 15 (8/10)	235 ± 13 (10/10)	206 ± 7 (10/10)	207.3	194.2
V	Beginning	16 Nov ± 5	14 Nov ± 7	03 Nov ± 9		
	End	6 May ± 9	2 May ± 10	13 May ± 6		
	Duration (1)	171 ± 12 (8/10)	169 ± 15 (8/10)	193 ± 8 (8/10)	178.0	141.0

(1) (Number of mares presenting winter ovarian inactivity/total number of mares).

(2) With only the mares presenting winter inactivity taken into account.

(3) With all the mares in the group taken into account.

* After an accidental fracture, one mare was removed from the experiment.

The number of luteal phases per year during the 3 years of the experiment were 14.2 ± 2.8, 7.6 ± 2.6 and 9.5 ± 2.8 in the WF, R and V group respectively and significantly differed, each year, between groups (p < 0.05; p < 0.0001, p < 0.0001 for 1^st^, 2^nd ^and 3^rd ^year respectively). The number of cycles was consistently higher in the WF group than in the other two groups (p < 0.05) which differed from one another only during the 2^nd ^year (p < 0.01).

The start, the end and the duration of winter inactivity were different for the 3 groups each year (p < 0.01, p < 0.01, p < 0.0001 for 1^st^, 2^nd ^and 3^rd ^year respectively). The date of mid-point of winter ovulatory inactivity did not differ and the mean for the 3 years was February the 6^th^. The WF group had a period of ovulatory inactivity beginning later than in R (p < 0.001) and V (p < 0.05) groups, ending earlier than in R (p < 0.01) and V (p < 0.01) groups and with a shorter duration than the R (p < 0.0001) and V (p < 0.01) groups. A significant difference was observed between R and V groups for the beginning (p < 0.05) but not for the end and borderline statistical significance regarding the duration (P = 0.055) of the winter inactivity. There was a significant year effect on the start and the end of the ovulatory inactivity (p < 0.05). For the start of the ovulatory inactivity, this difference is due to second winter which differ from the first and third winter (P < 0.05). For the end of the ovulatory inactivity, it is the date of the 3^rd ^year which is slightly later than the 2 others (P < 0.05).

In the R group, during the 1^st ^winter, 2 mares showed continuous reproductive activity. These 2 mares were the fattest of this group at the beginning of the experiment and, by the end of the first autumn had lost only 14% of their fat thickness. In contrast, the 8 others lost 40% during the first 6 months of the experiment. Winter inactivity occurred in these 2 R mares only during the 2^nd ^and 3^rd ^winter when they had lost 23% of initial body weight, at the end of the second autumn.

During the 2^nd ^and 3^rd ^winters, when body weight was stable in WF and R groups, the start, the end and the duration of ovarian inactivity were highly correlated. In this way, the correlation for the 3^rd ^winter are: r = -0.52 (P < 0.01), r = -0.79 (P < 0.0001), r = 0.94 (P < 0.0001) for the start and the end, the start and the duration, the end and the duration respectively. The correlations between fat thickness and the start, end and duration of winter inactivity are presented in Table 4.

**Table 4:** Coefficients of correlation between the mean of fat thickness measured each 2 weeks during the season preceding winter ovarian inactivity, of mares and some parameters of ovarian inactivity.

Seasons	2^nd ^ovarian winter inactivity			3^rd ^ovarian winter inactivity		
	Beginning	End	Duration	Beginning	End	Duration
Spring	0.80	-0.49	-0.75	0.80	-0.64	-0.81
Summer	0.78	-0.47	-0.74	0.80	-0.64	-0.80
Autumn	0.76	-0.46	-0.74	0.79	-0.62	-0.79
Winter	0.73	-0.49	-0.72	0.70	-0.50	-0.69

(All these coefficients are significant at P ≤ 0.01 n = 29).

### Patterns of melatonin, glucose, insulin, GH, IGF-1 and leptin

**Figure 3:**
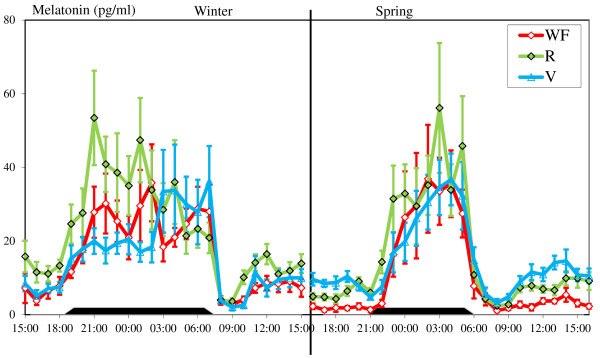
**Variations in plasma concentrations of melatonin**. Melatonin variations are shown over a 24 h period at two different times of year (end of winter and end of spring in WF, R and V groups, means ± S.E.M, one sample each hour). The V group was fed in winter like the WF group and in spring like the R group. The black rectangles indicate the natural night.

**Figure 4:**
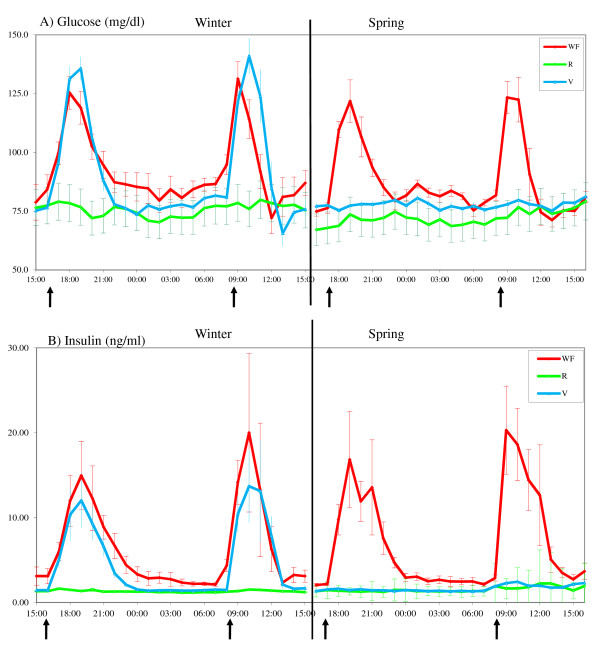
**Variations in plasma concentrations of glucose (A) and insulin (B)**. These variations were over a 24 h period at two different times of year (end of winter and end of spring in WF, R and V groups, means ± S.E.M, one sample each hour). The V group was fed in winter like the WF group and in spring like the R group. The arrows indicate the feeding times.

**Figure 5:**
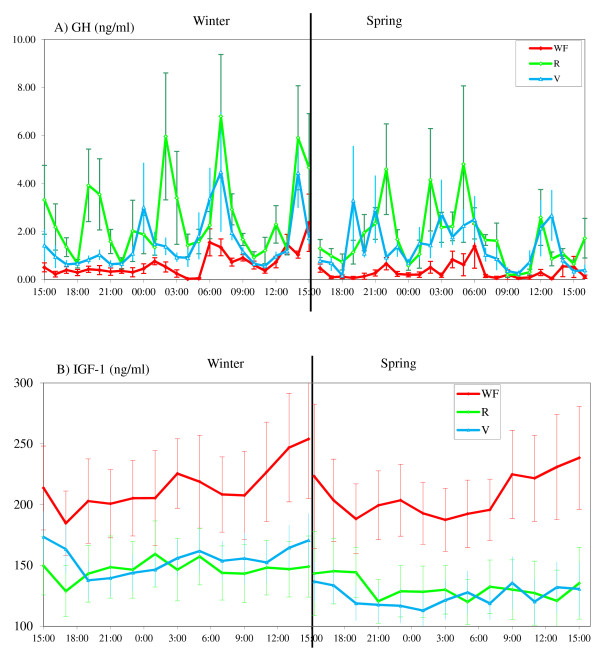
**Variations in plasma concentrations of GH (A) and IGF-1 (B)**. These variations were over a 24 h period at two different times of year (end of winter and end of spring in WF, R and V groups, means ± S.E.M, one sample each hour for GH, each 2 hours for IGF-1). The V group was fed in winter like the WF group and in spring like the R group.

**Figure 6:**
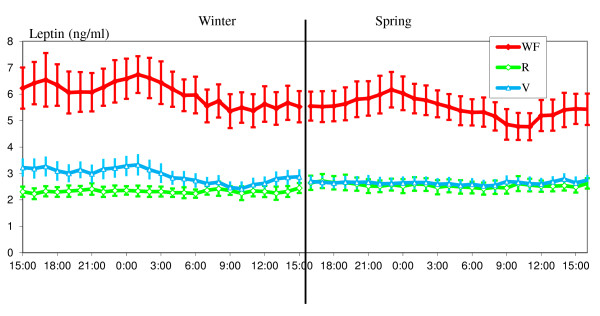
**Variations in plasma concentrations of leptin**. Leptine variations are shown over a 24 h period at two different times of year (end of winter and end of spring in WF, R and V groups, means ± S.E.M, one sample each hour). The V group was fed in winter like the WF group and in spring like the R group.

Plasma melatonin, glucose, insulin, GH, IGF-1 and leptin concentrations in the 3 groups during the two 24 h sampling periods at the end of winter and in the spring are shown in Figures 3, 4, 5 and 6.

*Melatonin: *Melatonin concentrations (Figure 3), measured under natural photoperiod, increased at dusk and decreased at dawn in all groups and these changes were significant for all the 3 groups (P < 0.01). No differences were found in the nocturnal melatonin level for all possible comparisons between groups or between seasons.

#### Glucose and insulin

Plasma concentrations of glucose and insulin (Figure 4) were different between groups (P < 0.0001). Glucose and insulin concentrations were affected by the period (defined in relation to feeding) and the interaction treatments - seasons was different (P < 0.0001). In the WF group, for both 24 h periods, and in the V group for the winter period when this group was fed similarly to the WF group, plasma glucose and insulin concentrations increased significantly after each meal (P < 0.0001 in comparison with similar periods in the R group). These increases were not significant in the R or in the V group, under restricted feeding (P > 0.54 within the R group and P > 0.69 within the V group under restricted feeding, in comparison with the daily period before meals). Concentrations returned to basal levels approximately 3 h after the start of the meal.

#### Growth hormone

The nutritional treatments had a significant effect on plasma GH concentrations (P < 0.0001) (Figure 5 A). These concentrations were greater (P < 0.01) in the R and V groups than in the WF group. The R and V groups had similar concentrations. The concentration of GH was higher in winter (P < 0.0001) but the interaction treatments - seasons was not statistically different.

#### IGF-1

The nutritional treatments had a significant effect on plasma IGF-1 (P < 0.05) (Figure 5 B). Concentrations were greater (P < 0.05) in the WF group than in the R and V groups which had similar concentrations. On plasma IGF-1, the season and the interaction treatments - seasons had a significant effect (P < 0.01). No difference was found on IGF-1 concentrations between winter and spring in WF or R groups but in the V groups this difference was significantly (P < 0.0001) higher during winter (feed intake as WF) than during spring (feed intake as R). The individual effect (nested under group) was also large (P < 0.0001).

#### Leptin

The nutritional treatments had a significant effect on plasma leptin level (P < 0.0001) (Figure 6). Concentrations were greater (P < 0.0001) in the WF group than in the R and V groups which had similar concentrations. The seasons and the interaction treatments - seasons had a significant effect on plasma leptin (P < 0.0001). In the V group, the decrease, from winter (well fed period) to spring (restricted period), was significant (P < 0.0001). Leptin concentrations showed significant variations with the light-dark cycle (P < 0.0001). Particularly, in the WF group plasma leptin was higher during the night than during the day in each season (P < 0.0001). However this was not the case in the R group. In the V group this difference was significant in winter (P < 0.01) but not in spring. During the 4 hours following each meal, the increase in plasma leptin concentration in comparison with the other times of day is significant only in the WF groups. In this group the levels during the morning or evening post-prandial periods are higher than during other times of day (P < 0.0001). No post-prandial effect was observed in the R group or in the V group (except in the R group in spring between the night level and the post-evening meal P < 0.05).

### Correlations among glucose, hormonal levels and duration of ovarian inactivity

The correlations between fat thickness and the different **glucose and hormonal **plasma levels are presented in Table 5. The fat thickness was significantly correlated with all the plasma hormone levels except, as expected, with melatonin which was not correlated with other hormone levels. The IGF-1 level was only correlated with insulin level.

**Table 5:** Correlations between fat thickness (mean of the measures taken during the winter of the blood sampling) and the different plasma levels (means of the 2 series of blood sampling), (Not Significant: NS; number of observations: 29)

	fat thickness	Melatonin	Glucose	Insulin	GH	IGF-1
Melatonin	NS					
Glucose	0.75 P < 0.0001	-0.44 P < 0.05				
Insulin	0.64 P < 0.001	NS	0.62 P < 0.001			
GH	-0.73 P < 0.0001	NS	-0.57 P < 0.01	-0.59 P < 0.001		
IGF-1	0.42 P < 0.05	NS	NS	0.50 P < 0.01	NS	
Leptin	0.75 P < 0.0001	NS	0.47 P < 0.01	0.53 P < 0.01	-0.63 P < 0.001	NS

The correlations between mean glucose, insulin, GH and leptin hormone levels measured in each of the 2 periods of blood sampling and the start, end and duration of winter anoestrus in the 2^nd ^and 3^rd ^winters were all significant (Table 6).

**Table 6:** Correlations between melatonin, glucose, insulin, GH, IGF-1 and leptin mean levels during the two 24 h periods and the beginning, end and duration of the previous or following winter ovarian inactivity.

	2^nd ^ovulatory winter inactivity			3^rd ^ovulatory winter inactivity		
	Beginning	End	Duration	Beginning	End	Duration
Melatonin	-0.37 P < 0.05	NS	NS	NS	NS	NS
Glucose	0.69 P < 0.0001	-0.60 P < 0.01	-0.67 P < 0.0001	0.60 P < 0.001	-0.60 P < 0.001	-0.68 P < 0.0001
Insulin	0.54 P < 0.001	-0.57 P < 0.01	-0.60 P < 0.001	0.45 P < 0.05	-0.61 P < 0.001	-0.62 P < 0.001
GH	-0.82 P < 0.0001	0.62 P < 0.001	0.78 P < 0.0001	-0.71 P < 0.0001	0.59 P < 0.001	0.72 P < 0.0001
IGF-1	NS	NS	NS	NS	NS	NS
Leptin	0.60 P < 0.001	-0.63 P < 0.001	-0.67 P < 0.0001	0.58 P < 0.001	-0.68 P < 0.0001	-0.73 P < 0.0001

(Not Significant: NS; number of observations: 29).

This study clearly demonstrates the effect of body condition on the duration of winter ovulatory inactivity in mares. On average, the mares in the WF group had a winter anovulatory period of approximately 40 days (mean duration when all mares in the groups are taken into account), while mares in the R group stopped cycling for more than 190 days. The number of cycles was double in the WF group in comparison with of the R group. For a given mare, when BCS was stable, the presence or absence of winter ovulatory inactivity as well as its timing (beginning or end) of this inactivity were highly reproducible. This is the first study systematically test possible correlations between different body condition parameters and the duration of winter inactivity. The importance of fat thickness on duration of anovulatory period had been demonstrated previously [14]. The cessation of ovulation activity was also observed in cows, goats and humans [64-67] when the loss of body weight was substantial.

In 8 mares from the V group, in which the life "*in natura*" was mimicked there was no difference with the R group in the duration of winter inactivity (except for the 2 mares which did not show any winter inactivity, probably because they did not lost enough body weight). The result from the V group indicates that the effect of feed intake on the end of winter inactivity does not depend from autumnal or winter period (period where the V group was fed as the WF group) but rather from previous and undetermined season. This phenomenon suggests in mares the existence of putative hypothalamic nutritional memory. Some mechanisms of this metabolic memory were previously showed in ram and seem to involve the Leptin/NPY system in the brain [68]. This absence of response to the change of feeding contradicts several studies conducted during spring in mares which are kept on pasture ([6,69,70]. In our study, protein quality was not taken into account, but it probably influences the beginning of the reproductive season [71]. The effect of feeding with high quality protein in spring on the beginning of reproduction looks like a flushing effect described in small ruminants [72].

In the 3 groups, the winter inactivity is centered on the same date at the beginning of February, so the timing of ovulatory inactivity is clearly governed by photoperiod. In all 3 groups, the observation 'for a given mare, later the last ovulation of the year, earlier the first ovulation of the following year' is supported [73]. In all 3 groups, the occurrence and the duration of winter ovulatory inactivity in each animal is very reproducible. This phenomenon implies a genetic and/or epigenetic control of the sensitivity to nutrition level on winter inactivity. In ram, this genetic effect was shown by the different effect of photoperiod and nutritional treatments on 2 breeds, Suffolk and Merinos [74]. In mares a breed effect was also observed on the end of winter inactivity; the first ovulation occurred in all the type of mares studied later in Finnhorse mares than in Warmblood mares [9]. This difference probably is the consequence of the selection on race ability of Thoroughbred horses (genes of this breed are generally present in all the warmblood mares). This selection on race ability has privileged some genes which had an effect on metabolic routes particularly on insulin receptor signaling [75]. The Welsh pony mares which were never selected on race ability until the last 2 decades seem to be a suitable model for wild horses. The epigenetic effect of a nutrition schedule, on the leptin system was recently demonstrated, in mice, by Jousse et al. [76]. This epigenetic effect could be another mechanism implicated in metabolic memory.

The melatonin assay, as expected, confirmed previous results: The daily secretion is associated with photoperiod so the pattern is longer in winter than in spring but the nocturnal plasma melatonin level is not changed by the treatment [77-79] and not associated with the occurrence of winter inactivity [80,81]. The lack of a significant correlation between the duration of winter inactivity and night melatonin level confirms that melatonin is not the neuro-hormone involved in the duration of winter inactivity. The low correlation found during the first winter is probably attributable to a slight difference in melatonin clearance between groups. The sole role of photoperiod or melatonin for winter inactivity is probably to synchronize a putative endogenous annual rhythm with the seasons.

In our study, plasma glucose and insulin concentrations during the two 24 h periods of blood sampling showed marked differences among the 3 groups. Glucose and insulin in the WF group had a post-prandial peak in both periods of sampling. This peak was not present in the R group. In the V group, it was present when the mares were fed as the WF group but not when they were fed as the R group. So, the rate of adjustment of glucose and insulin variations to the feed intake concur with the study of Sticker *et al*. [82]. The importance of glucose for the hypothalamic release of GnRH or LH is now well demonstrated in different mammalian species [83-88]. Insulin, which has receptors in the hypothalamus [89], seems to be also important for LH release [90]; but in equine species, this effect is controversial [91,92]. A long-term effect of glucose or insulin on the anovulatory period is unclear because the glucose or insulin concentrations, in monogastric species, adapts spontaneously to the levels to feed intake. So, glucose or insulin levels provide a variable message which is not compatible with the stability of the duration of winter inactivity. This stability observed in the V group suggests that glucose or insulin have no direct effect on the mechanism of nutrition on reproduction.

Our results shows that in horses, low plasma glucose and insulin concentrations are associated with elevated GH secretion in the R group. Hypoinsulinemia may be responsible for the specific low sensitization of GH receptors in the liver of restricted animals [93], then the plasma levels of IGF-I are reduced and the negative feedback of IGF-I on the somatotropic axis is diminished [94]. However, in our experiment, the IGF-1 plasma level had a low and non-significant correlation with GH or leptin level (r = -0.24 NS; r = 0.20 NS) but a significant correlation with the insulin level (r = 0.50 P < 0.01). Then, the major involvement of insulin in IGF-1 secretion is suggested by our data.

In horses, Buff *et al*. [24] found a strong correlation between the plasma levels of leptin and BCS (r = 0.64, P < 0.0001). This correlation is confirmed in our study with a specific assay (correlation with the means, of all the measures of fat tissue thickness or with BCS taken during the winter of the blood sample collection, r = 0.75, P < 0.001, r = 0.78, P < 0.001 respectively). In a previous experiment, using treatment with a Beta2 adrenergic agonist with anabolic effect, Clenbuterol, McManus and Fitzgerald [42] induced a decrease in the fat mass and consecutively on leptin level. This treatment acts also probably on other adiponectins levels involved in the winter anovulatory. This treatment induced a long winter ovulatory inactivity. The existence of a daily rhythm of leptin secretion in fat horses (BCS between 3 and 3.5) previously described [95] is confirmed in fat mares but not in thin ones. The correlation between plasma concentration of leptin and the duration of winter inactivity is in accordance with the major role of this hormone as a messenger of metabolic status for the central regulation.

## Conclusion

We can draw conclusions about the respective influences of nutrition and photoperiod on the annual rhythm of reproduction in mares. Ovulatory activity of the mare represents the visible part of the annual rhythm. The timing of this rhythm in relation to the season is determined by photoperiod through the melatonin pattern. The higher the energy level available for ovulatory activity, the longer the phase of ovulatory activity. However, the optimum timing of the rise in energy store to maintain ovulatory activity is not defined. The neuro-endocrine mechanisms of this nutrition-photoperiod interaction may involve the leptin-GH-IGFs systems.

## List of abbreviations

BCS: Body condition score; GH: Growth Hormone, also known as somatotropin; GLM: general linear model procedure: a type of analyse of variance in SAS software; GnRH: Gonadotropin releasing hormone; IGF-1: Insulin like Growth Factor 1; LH: Luteinizing Hormone; MADC: Horse Digestible Crude Protein (INRA 1997); R: experimental group of mares with Restricted feed intake; RIA: Radio-Immuno-Assay; UFC: Horse Feed Unit (INRA 1997); V: experimental group of mares with Variable feed intake some time as WF group some time as R group; WF: experimental group of Well Fed mares with feed intake calculated to be in good body condition.

## Competing interests

The authors declare that they have no competing interests.

## Authors' contributions

JSO has supervised the entire work one the animals and done the glucose, insulin and GH assays. SC has developed, validated and done the leptin assays. CB has done the non-parametric test. LL has done the IGF1 assays. DC has done the melatonin assays. DG has supervised all the work, developed and validated the GH assay, done the statistical analysis with SAS and writes the manuscript. All authors read and approved the final manuscript.

## Authors' information

JSO during the experiment was PhD student. He is now "Profesor Investigador Asociado" at the "Colegio de Postgraduados" Campus Córdoba MEXICO. SC is a permanent research worker. CB was a permanent research worker. LL is a technical assistant of the staff. DC is a technical assistant of the staff. DG is a permanent research worker.
